# Porous Coordination Polymer MOF-808 as an Effective Catalyst to Enhance Sustainable Chemical Processes

**DOI:** 10.3390/polym16070968

**Published:** 2024-04-02

**Authors:** Catarina E. S. Ferreira, Isabel Santos-Vieira, Carlos R. Gomes, Salete S. Balula, Luís Cunha-Silva

**Affiliations:** 1 LAQV/REQUIMTE & Department of Chemistry and Biochemistry, Faculty of Sciences, University of Porto, 4169-007 Porto, Portugal; 2 CICECO—Aveiro Institute of Materials, Department of Chemistry, University of Aveiro, 3810-193 Aveiro, Portugal; ivieira@ua.pt; 3 CIMAR/CIIMAR—Centro Interdisciplinar de Investigação Marinha e Ambiental & Faculdade de Ciências, Universidade do Porto, 4169-007 Porto, Portugal; crgomes@fc.up.pt

**Keywords:** porous coordination polymers, metal–organic framework, MOF-808, heterogeneous catalysts, epoxides ring opening, sustainable processes

## Abstract

The improvement of sustainable chemical processes plays a pivotal role in safe environmental and societal development, for example, by reducing the use of hazardous substances, preventing chemical waste, and improving the efficiency of chemical reactions to obtain added-value compounds. In this context, the porous coordination polymer MOF-808 (MOF, metal–organic framework) was prepared by a straightforward method in water, at room temperature, and was unequivocally characterized by powder X-ray diffraction, vibrational spectroscopy, thermogravimetric analysis, and scanning electron microscopy. MOF-808 material was applied for the first time as catalysts in ring-opening aminolysis reactions of epoxides. It demonstrated high activity and selectivity for reactions of styrene oxide and cyclohexene oxide with aniline, using a very low amount of an eco-sustainable solvent (0.5 mL of EtOH), at 70 °C. Moreover, MOF-808 demonstrated high stability in the catalytic reaction conditions applied, and a notable reuse capacity of up to 20 consecutive reaction cycles, without significant variation in its catalytic performance. In fact, this Zr-based porous coordination polymer prepared by environment-friendly conditions proved to be a novel efficient heterogeneous catalyst, promoting the ring-opening reaction of epoxides under more sustainable conditions, and using a very low amount of catalyst.

## 1. Introduction

Metal–organic frameworks (MOFs) are a class of crystalline materials consisting of metal centers (nodes) interconnected by organic ligands (linkers) via coordination bonds, ultimately forming porous three-dimensional (3D) porous coordination polymers [1]. In the last few years, numerous studies have shown that MOFs can overcome the potential of other known porous solid materials such as zeolites and porous carbon-based materials in a variety of applications [2]. In fact, the scientific interest in these materials is due to the huge variety of organic and inorganic components used for the preparation of MOFs originating large structural variety and the easy introduction of functional groups in the ligand, enhancing MOFs for several applications and areas of interest such as gas storage, adsorption, luminescence, sensors, catalysis, therapy for various diseases, such as cancer, water/air purification, and others [1,3,4,5,6,7].

The use of MOFs as heterogeneous catalysts was one of the most promising applications, along with its rapid development in the last twenty years [8]. Because of the enormous possibility of combining various active catalytic sites (active metal centers and/or active functional organic linkers) in the same material, porous MOFs have attracted significant scientific interest as heterogeneous catalysts in a wide range of chemical reactions [9,10,11,12,13,14]. In general, the structures of MOFs reveal active sites uniformly dispersed throughout the framework, and the characteristic porosity of MOFs tends to facilitate the transport of substrates and products and to facile access of the catalytic active sites. Frequently, MOFs present highly active and stable structures, which can possibly be recycled or reused in up to several reaction cycles. In fact, some MOFs can demonstrate high catalytic performances as homogeneous catalysts and also the capacity for reutilization that is characteristic of heterogeneous catalysts [8,15,16]. In particular, Zr(IV)-based MOFs with carboxylate-type ligands, for example, MOF-808, tend to be formed by the Zr–O cluster interconnected by the several mentioned ligands. These peculiar structural features provide Zr-based MOFs with interesting characteristics, such as high (nano)porosity, excellent thermal and hydrolytic stability, high specific surface area, and the possibility to accommodate framework defects, giving them enormous catalytic potential as heterogeneous catalysts [17,18,19]. Specifically, MOF-808 is formed by clusters [Zr_6_O_4_(OH)_4_]^12+^ like those present in the well-known UiO-66, but each cluster is connected only by six trimesate ligands, and the other coordination positions of the Zr cations are saturated by ion-shaped molecules.

**Figure 1:**
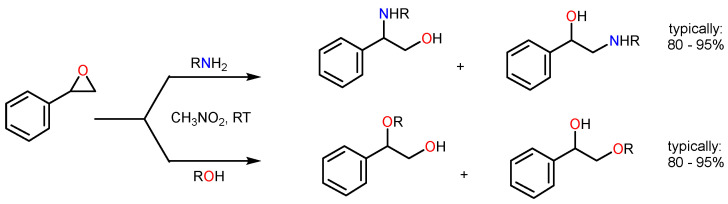
Scheme of the epoxide ring-opening reaction, both by amines and alcohols, using nitromethane as a solvent at room temperature; the products represented are usually the majority (with yields usually ranging from 80% to 95%).

The ring-opening reactions of an epoxide by amines have relevant importance since the products of this reaction, such as β-amino alcohols, have an extraordinary interest in the preparation of biologically active and synthetic compounds, as well as other neutral products, such as chiral pharmaceutical molecules, insecticides, and others [20,21,22,23] (Figure 1). β-amino alcohols are generally synthesized by a direct reaction of epoxide aminolysis. Epoxides are one of the most versatile intermediates used in organic synthesis and react with a wide variety of reagents such as electrophiles, nucleophiles, acids, bases, reducing agents, and some oxidizing agents [24]. These present an easy-to-reproduce synthesis due to ring deformation and still react with different nucleophiles with high regioselectivity, originating products that have an open ring [25]. The fact that there is a high reactivity with several nucleophiles produces highly regioselective and trans-stereospecific ring-opening products. Therefore, there is currently a great interest in the study of opening reactions of the epoxy ring [26,27]. However, this reaction most often involves a large excess of amines, and the use of other extreme reaction conditions, such as elevated temperatures, drastically reduces its industrial attractiveness. The cost-efficiency and sustainability of this type of process can be improved using efficient, selective, and recyclable solid catalysts.

**Figure 2:**
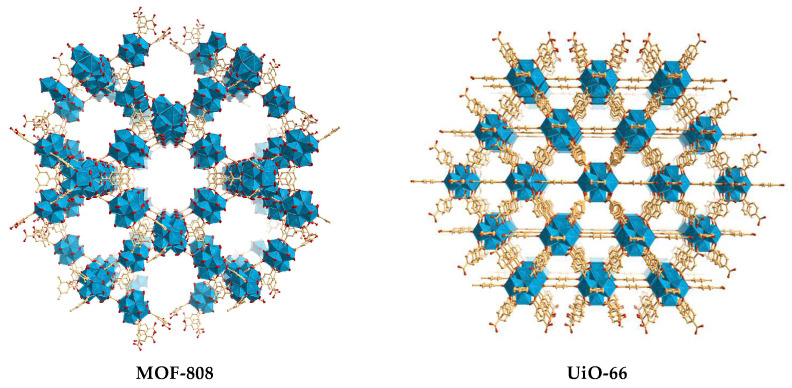
Crystalline structures of the porous MOF materials, MOF-808 and UiO-66 (identical to UiO-66-NH_2_), revealing the same metal cluster center; the images were prepared from CIF files obtained from the Cambridge Structural Database (CSD, reference codes BOHWUS [37] and AZALUL [38], respectively).

Most of the catalysts reported for these types of reactions are homogeneous and thus, are practically impossible to reuse, and they are often expensive, inefficient, toxic, or unstable. For this reason, it is important and fundamental to evaluate unprecedented materials as sustainable heterogeneous catalysts [28,29]. Following the research interest of our research group in the development of MOF-based materials towards different sustainable catalytic processes [30,31,32,33,34,35,36], MOF-808 was prepared by a greener procedure, properly characterized, and evaluated as a catalyst in two ring-opening catalytic reactions, one of styrene oxide and the other of cyclohexene oxide (another Zr-based porous coordination polymer, UiO-66-NH_2_, was also prepared and applied for comparative proposes; Figure 2). MOF-808 proved to be an efficient heterogeneous catalyst for this type of reaction.

## 2. Materials and Methods

### 2.1. Reagents and Products

Styrene oxide (C_8_H_8_O, 97%), cyclohexene oxide (C_6_H_10_O, 98%), aniline (C_6_H_7_N, 99%), and trimesic acid (C_9_H_6_O_6_, 95%) were acquired from Aldrich. The phosphomolybdic acid hydrate (H_3_PMo_12_O_40_.xH_2_O) was acquired from Sigma-Aldrich. Formic acid (CH_2_O_2_, 90%) and dimethylformamide (C_3_H_7_NO, ≥99.5%) were acquired from Fisher. Acetonitrile (CH_3_CN, 99.5%, MeCN), acetone (C3H6O, 99.8%), and methanol (CH_4_O, ≥99.9%) were purchased from Carlo Erba. Zirconium chloride (ZrCl_4_, 99.5%) was acquired from Alfa Aesar. Ethanol (C_2_H_6_O, ≥99.8%) and acetic acid (C_2_H_4_O_2_, ≥99.8%) were purchased from Honeywell. Isopropanol (C_3_H_8_O) was acquired from Merck Millipore. These chemical compounds used in this research work were used as received, without any type of purification treatment prior to use.

### 2.2. Characterizations Methods

Powder X-ray diffraction (PXRD) patterns were collected at room temperature on an Empyrean PANalytical diffractometer (CuKα1/2 radiation, λ1 = 1.540598 Å, and λ2 = 1.544426 Å), equipped with a PIXcel 1D detector and a flat-plate sample holder in a Bragg–Brentano para-focusing optics configuration (45 kV, 40 mA); intensity data were obtained by the step-counting method (step 0.02°), in continuous mode in the approximate range of 3.0° ≤ 2θ ≤ 50°. Infrared spectra were recorded on a PerkinElmer FT-IR System Spectrum BX spectrometer (in the range of wavenumbers: 400 to 4000 cm^−1^ and 64 scans). FT-Raman spectra were recorded in a Bruker spectrometer RFS 100, using as excitation source a Nd: YAG laser (λ = 1064 nm) at room temperature, in the frequency range of 3600–50 cm^−1^, with a resolution of 4 cm^−1^ (the excitation power and number of steps were selected according to the sample). Scanning Electronic Microscopy (SEM)/Energy-Dispersive X-Ray Spectroscopy (EDS) analyses were performed at the “Centro de Materiais da Universidade do Porto” (CEMUP) using an Environmental Scanning Electron Microscope, high resolution (Schottky), with X-ray Microanalysis and Analysis of Electron Diffraction Patterns: FEI Quanta 400FEG ESEM/EDAX Genesis X4M; the samples were coated with Au/Pd thin film by cathodic pulverization (sputtering) using the SPI Module Sputter Coater equipment. Thermogravimetric analyses (TGAs) were performed using a thermobalance Thermal Analysis system STA 300 Hitachi, using a heating speed of 5 C/min and an N_2_ atmosphere. Inductively coupled plasma optical emission spectrometry (ICP-OES) tests were performed at the University of Santiago de Compostela, Spain, on PerkinElmer Optima 4300 DV equipment. The quantification of the products of the two ring-opening reactions along the catalytic ring-opening reactions was performed in a Scion 8300 GC gas chromatograph, using hydrogen as drag gas, with a linear velocity of 55 cm^3^.s^−1^ and using a capillary column of SPB-5 Supelco (30 m long, 250 μm inner diameter and 25 μm film thickness); the total time per analysis was 18 min. Gas chromatography coupled to mass spectrometry (GC-MS) was performed in a Thermo Scientific Trace 1300 chromatograph coupled to a Thermo Scientific ISQ Single Quadruplo MS device. In both cases, TG-5MS columns (30 m; 0.25 mm (i.d.); 0.25 µm) were used.

### 2.3. Preparation of the Materials

The preparation of the oxoclusters and, posteriorly, the synthesis of MOF-808 with three different particle sizes was performed following procedures previously reported in the literature [39]. The synthesis at room temperature of this MOF was carried out using pre-formed octahedral oxoclusters Zr_6_, which is the most common SBU in the family of MOFs based on Zr (IV) with the carboxylate functional ligands.

Oxoclusters. A total of 25.17 g (0.11 mol) of ZrCl_4_ was mixed with 37.5 mL of acetic acid and 62.5 mL of isopropanol, and the mixture was stirred until a homogeneous solution was obtained. The solution was heated in a paraffin bath at 120 °C for 1 h, and the white suspension obtained was then centrifuged and washed twice with acetone. Finally, the material was dried by heating (60 °C) at reduced pressure (200 mbar), and the white powder obtained was characterized by FTIR-ATR.

MOF-808. A total of 0.6 g of oxoclusters was added to 1.5 mL of formic acid, and the solution was stirred at room temperature. Then, 2.5 mL of H_2_O was added, and the solution changed from whitish to transparent, as reported in the literature [39]. Finally, 150 mg (0.7 mmol) of trimesic acid was added, and the reaction mixture was kept in agitation overnight. The material obtained was isolated by centrifugation, washed twice with H_2_O and ethanol, and dried by heating (60 °C) at reduced pressure (200 mbar). Yield (%): 73.8.

UiO-66-NH_2_. The synthesis procedure followed the solvothermal method adapted from the literature [40]. Briefly, 2.17 g (0.012 mol) of 2-aminoterafthallic acid and 3.8 g (0.016 mol) of ZrCl_4_ were mixed in 36 mL of DMF. The mixture was stirred for 30 min and inserted in autoclaves that were placed in the oven at 120 °C for 24 h. A yellow solid was isolated by centrifugation, washed with DMF and methanol, and dried by heating at 70 °C and reduced pressure (under vacuum). Yield (%): 82.5.

### 2.4. Catalytic Studies

**Figure 3:**
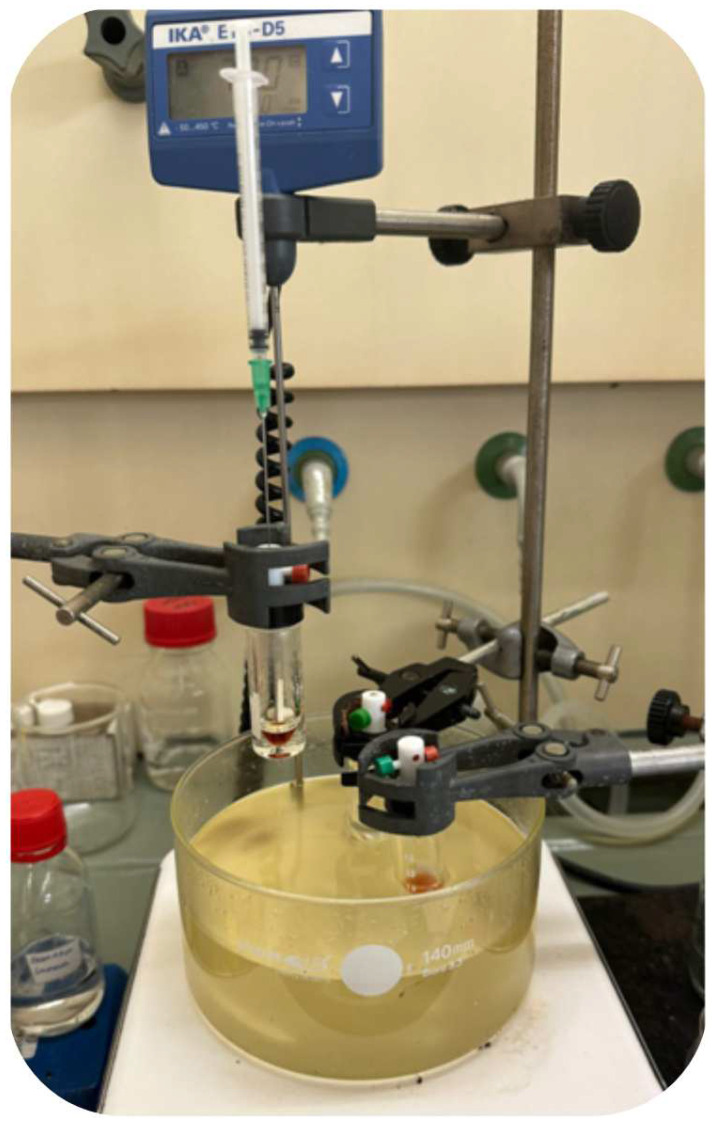
Experimental apparatus used to perform the catalytic experiments.

The ring-opening reactions of styrene oxide (1 mmol) and cyclohexene oxide (1 mmol) with aniline (0.9 mmol) were carried out in a borosilicate 5 mL reaction vessel with a magnetic stirrer and immersed in a thermostat oil bath under air (atmospheric pressure), using an amount of catalyst (10 mg) containing 1 μmol of Zr and 1.5 mL of acetonitrile (MeCN) or ethanol (EtOH) as a solvent, at 70 °C (Figure 3). The recycling study of the catalyst was performed by using the same portion of the solid catalyst during various consecutive cycles, dispersed in 0.5 mL of EtOH, and after each catalytic cycle, the catalyst was recovered by centrifugation, washed with EtOH (twice), and dried. The recovered MOF was weighed and reused in a new catalytic cycle maintaining the experimental conditions. Further, the reutilization ability of the catalyst was also studied, and in this case, the catalyst was not washed or dried after each catalytic cycle. The catalyst was used without treatment between cycles, maintaining all the experimental conditions. All the reactions were monitored by periodic GC analysis taking an aliquot from the reaction mixture at regular intervals until the product yields remained constant during at least 2 h of reaction. The products were analyzed by GC/GC–MS techniques. Moreover, the recovered catalysts were characterized by powder XRD and FT-IR spectroscopy after catalytic use.

## 3. Results and Discussion

### 3.1. Preparation and Characterization of the Catalysts

**Figure 4:**
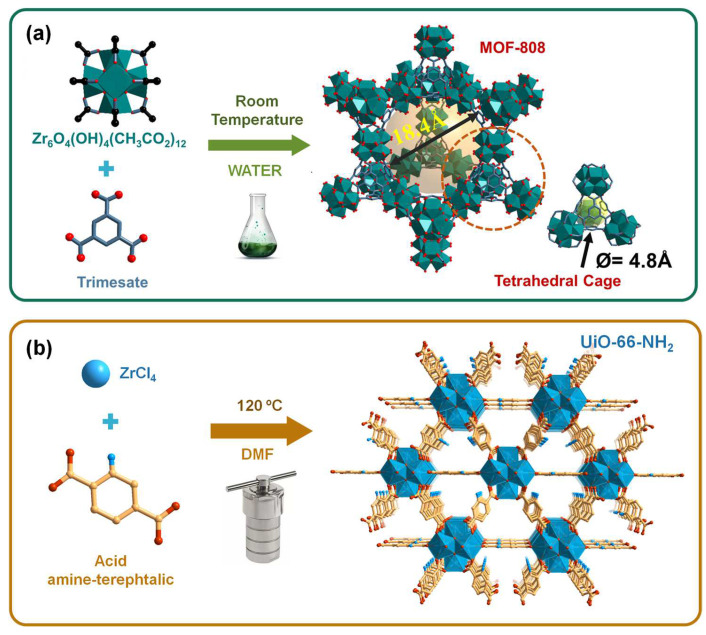
(**a**) Schematic representation of the sustainable synthesis of MOF-808 (in water and at room temperature) and (**b**) the scheme of the preparation of UiO-66-NH_2_, showing some features of the crystalline structure of the porous coordination polymers. Adapted from reference [39].

The syntheses of Zr-based MOFs are usually performed by the solvothermal procedure using organic solvents, such as DMF, with some negative environmental impact. However, nowadays, it is crucial to obtain these MOF structures using more sustainable synthesis routes, especially under aqueous conditions and/or at room temperature. The research in the context of new conditions of green synthesis is an important focus in the area of MOF materials, especially motivated by the transition to an industrial-scale synthesis that would be practically impossible with the use of hazardous chemicals under adverse reaction conditions [41]. In this work, while the MOF UiO-66-NH_2_ was prepared by the conventional solvothermal procedure (high temperature and DMF as a solvent), MOF-808 was prepared by a more sustainable method (room temperature and water as a solvent; Figure 4). Both porous MOFs, i.e., MOF-808 and UiO-66-NH_2_, were further characterized by a myriad of techniques including vibration spectroscopies (FTIR and FT-Raman), powder XRD, TGA, elemental analysis (ICP), scanning electron microscopy (SEM), and energy-dispersive X-ray spectroscopy (EDS), confirming the preparation of the expected solid-state pure phases of the two porous MOF materials. The porous nature of these coordination polymers is well documented in the literature (MOF-808: BET area ~1600 mg^2^cm^−1^, specific volume 0.69 cm^3^g^−1^, pore size 18.4 Å; UiO-66-NH_2_: BET area ~870 mg^2^cm^−1^, specific volume 0.38 cm^3^g^−1^, pore size 9.5 Å).

**Figure 5:**
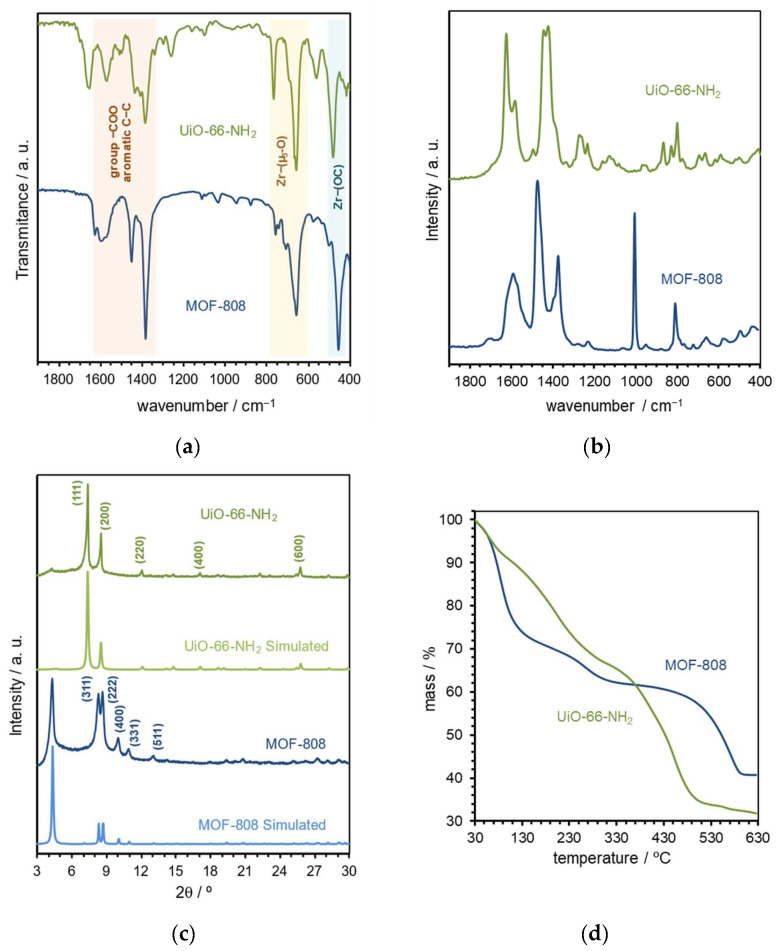
Selected characterization data for the prepared MOF materials, MOF-808 (blue color) and UiO-66-NH_2_ (green color): (**a**) FT-IR and (**b**) FT-Raman showed in the wavenumber range from 1900 to 400 cm^−1^; (**c**) simulated and experimental powder XRD patterns (the simulated diffractograms were obtained from the respective crystallographic data deposited in the Cambridge Structural Database); and (**d**) TGA profiles showed in temperature range of 30–630 °C.

The FT-IR and FT-Raman spectra obtained for both the Zr-based MOF-based materials reveal the main vibrational band characteristics as expected from the two MOF structures (Figure 5a,b; spectra shown in the 1900–400 cm^−1^ wavenumber region) [42,43]. Briefly, the medium and strong intensity bands to vibrational modes of the carboxylate groups can be assigned from 1600 to 1390 cm^−1^; a vibrational band around 1450 cm^−1^ ascribed to aromatic (C–C) bonds of the organic ligands; as well as a group of vibrational bands associated with Zr–(μ_3_-O) framework bonds in the range of 800–600 cm^−1^, and a band (FT-IR) around 450 cm^−1^ assigned to Zr–(OC) bonds. These vibrational spectra are comparable with those previously reported, being the first clear indication of the preparation of the desired MOF materials, MOF-808 and UiO-66-NH_2_. This fact is completely confirmed by the powder XRD patterns of the isolated materials that reveal the characteristic reflections of the MOF-808 and UiO-66-NH_2_ crystalline phase, both in location and relative intensities (Figure 5c): in the diffractogram of MOF-808, the main 2Ɵ diffraction peaks at the 4.3°, 8.3°, 8.7°, 10.0°, 11.0°, and 13.0° plans are assigned to the reflection plans (111), (311), (222), (400), (331), and (511), respectively; for the UiO-66-NH_2_ diffraction pattern, the main reflections at 7.4°, 8.5°, 11.9°, 14.6°, 16.8°, and 25.6° are attributed to the plans (111), (200), (220), (222), (400), and (600), respectively. Furthermore, the powder XRD analysis also allows for discarding the coexistence of any secondary crystalline phases in the two isolated materials by comparison with the diffractogram obtained from the crystallographic data. In fact, the experimental diffractograms prove the preparation of both the MOF-808 and UiO-66-NH_2_ materials with the expected solid-state structure [39,44]. As expected, the thermogravimetric analysis profiles of the two materials are like those previously reported and confirm the thermal stability of MOF-808 up to about 500 °C and up to 400 °C for UiO-66-NH_2_ (Figure 5d) [45,46].

**Figure 6:**
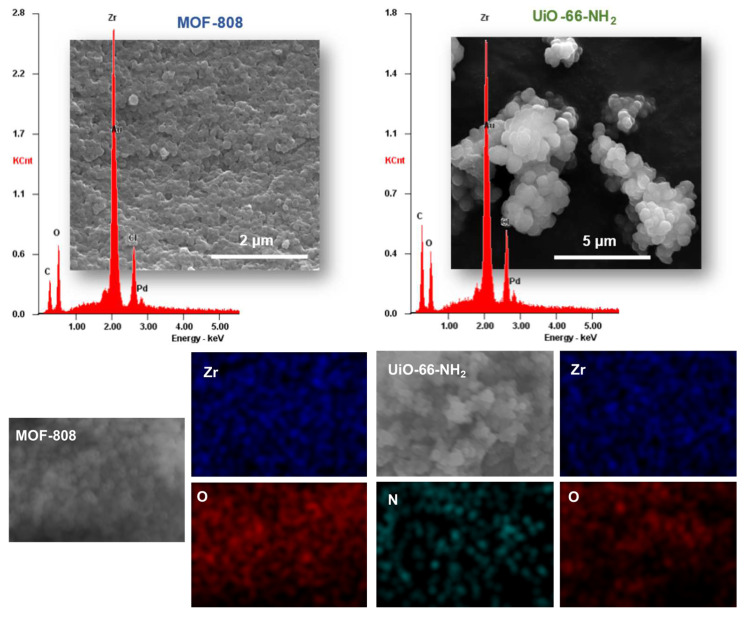
SEM images, EDS spectra, and elemental mappings for the prepared porous coordination polymers, MOF-808 and UiO-66-NH_2_.

The micrographs obtained by SEM for the two materials in random zones of the samples reveal agglomerated particles, apparently with superior crystalline regularity in the UiO-66-NH_2_ sample, most probably because of its solvothermal preparation instead of the room temperature preparation of MOF-808 (Figure 6). In addition, the EDS analyses clearly demonstrate the presence of the following characteristic elements of the MOFs: Zr, O, and C (also N for UiO-66-NH_2_). The EDS elemental mappings confirm and homogenous distribution of the main elements of the material structure. The combination of all the data obtained by several characterization techniques confirms the successful preparation of the two Zr-based porous MOF materials, MOF-808 and UiO-66-NH_2_.

### 3.2. Catalytic Studies

The prepared Zr-based materials were evaluated as heterogeneous catalysts for ring-opening reactions of epoxides, in particular, styrene oxide and cyclohexene oxide, by amines (aniline). The influence of some experimental reaction conditions was initially investigated, namely, the nature and the volume of the used solvent. This study intends to use more sustainable solvents at the lowest amount needed that can guarantee the highest catalytic activity of the catalytic materials. Blank experiments were conducted without the presence of the MOF catalysts using both epoxide substrates (Table 1). The results indicate that practically no conversion of substrates was observed in the absence of the catalyst, given that the highest conversion under these conditions was less than 16% for styrene oxide, after 24 h using 0.5 mL of ethanol (EtOH).

**Figure 7:**
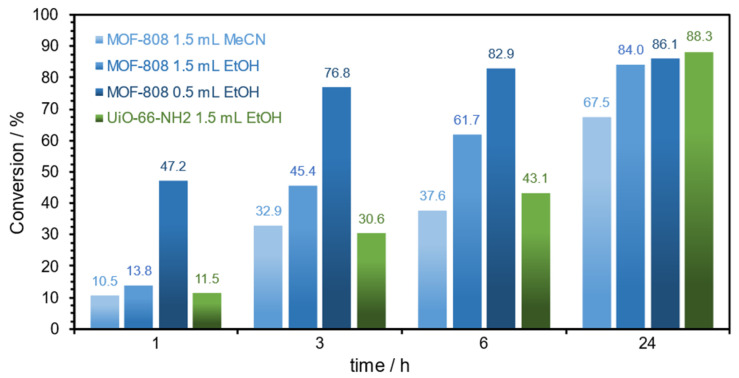
Conversion data for the ring-opening reaction of styrene oxide (1 mmol) in the presence of aniline (0.9 mmol), using MOF-808 as a catalyst (1.5 mL of MeCN and 1.5 mL and 0.5 mL of EtOH as solvents), and UiO-66-NH_2_ as a catalyst (1.5 mL of EtOH as a solvent), at 70 °C.

The Zr-based MOF catalysts, MOF-808 and UiO-66-NH_2_, were then used as heterogeneous catalysts. The initial experimental conditions used were based on the previously published work from our research group using the following Fe-based MOFs: MIL-101(Fe) and MIL-101(Fe)-NH_2_, i.e., 1 mmol substrate, 0.9 mmol of aniline, 1.5 mL of MeCN, and 70 °C temperature [28]. The results obtained for the conversion of styrene oxide are presented in Figure 7. It is possible to observe that during the first 6 h of the reaction, MOF-808 showed to be more active than UiO-66-NH_2,_ mainly using EtOH as solvent instead of MeCN. After 6 h of reaction, 82.9% of styrene oxidation was obtained using MOF-808 as a catalyst and a very small amount of the sustainable solvent EtOH (0.5 mL), instead of 61.7% obtained with a higher EtOH amount (1.5 mL), and 37.6% of conversion using MeCN (1.5 mL). After 24 h, the conversion increased to 86.1% using MOF-808 and 0.5 mL of EtOH. When UiO-66-NH_2_ was used, a similar conversion was achieved (88.3%) using 1.5 mL of EtOH. From these results, it was possible to conclude that a higher conversion was possible to be obtained with a lower amount of solvent used. Therefore, future experiments were performed with EtOH instead of MeCN.

**Figure 8:**
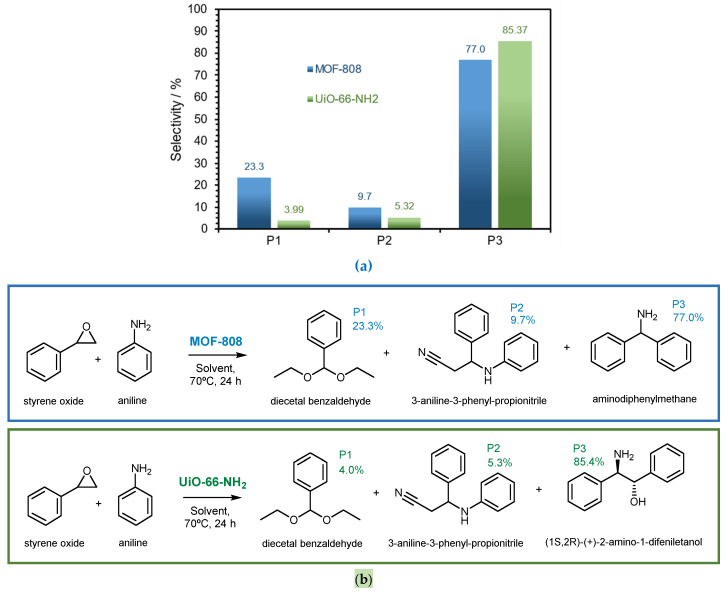
(**a**) Selectivity obtained for the ring-opening reaction of styrene oxide with aniline in the presence of MOF-808 (blue) and UiO-66-NH_2_ (green) as catalysts showing the 3 main products (P1, P2, and P3) (24 h; a temperature of 70 °C and EtOH as a solvent). (**b**) Reaction schemes using the two MOFs as catalysts showing the respective obtained products (P1, P2, and P3).

In addition to the interesting catalytic activity of the two Zr-based MOFs for the conversion of styrene oxide with aniline, both the MOF-808 and UiO-66-NH_2_ catalysts also showed high selectivity, obtaining amino derivative main products (Figure 8). Under the studied conditions, both MOF catalysts promoted, essentially, the production of a distinct main product (77% of aminodiphenylmethane with MOF-808 and 85% of (1S,2R)-(+)-2-amino-1,2-diphenyllethanol with UiO-66-NH_2_) and two minor identical products (diecetal benzaldehyde and 3-aniline-3-pheny propionitrile), as presented in Figure 8b. The various products obtained in the catalytic reactions were identified by GC-MS.

The use of Zr-based MOFs as catalysts did not originate β-amino alcohols as products. The mechanism to obtain these is well-known in the literature and consists of two main steps. The reaction may be divided into the following two steps: (i) the epoxide ring-opening by the interaction of the nitrogen from the aniline with one of the two carbons from the epoxide and (ii) a transfer from a proton from aniline to the alkoxide oxygen atom to yield the β-amino alcohol product [22]. The studied Zr-based MOFs can have easily coordinated vacancies to the Zr metal center, which can interact easily with the epoxide, modifying the nature of the obtained products.

**Figure 9:**
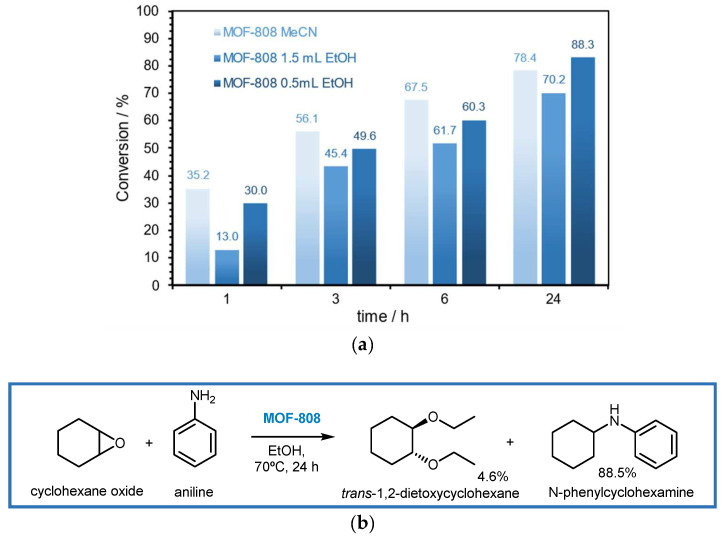
(**a**) Result obtained for the catalytic reaction of the ring opening of cyclohexene oxide (1 mmol) with aniline (0.9 mmol), using the MOF-808 catalyst and 1.5 mL of MeCN, and 1.5 mL and 0.5 mL of EtOH as solvents, at 70 °C. (**b**) Scheme of the reaction of the ring opening of cyclohexene oxide with aniline using the MOF-808 catalyst and 0.5 mL of EtOH.

The potential of MOF-808 as heterogeneous catalyst in the ring-opening reaction of epoxides with amines was further validated with a second distinct molecule, cyclohexene oxide. The experimental conditions of the reaction were the same as those used for styrene oxide (1 mmol of epoxide substrate, 0.9 mmol of aniline, catalyst, at 70 °C). The nature and the amount of the solvent were also here studied; therefore, 1.5 mL of MeCN and 1.5 mL and 0.5 mL of EtOH were used. The results are presented in Figure 9a, which reveal that the conversion of cyclohexene oxide after 24 h was 78.4% (1.5 mL MeCN), 70.4% (1.5 mL EtOH), and 88.3% (0.5 mL EtOH). As in the previous study with styrene oxide, also using cyclohexene oxide as substrate, the use of EtOH as solvent originated the highest conversion. This was achieved using low volume of this solvent, i.e., 0.5 mL of EtOH), where the the conversion obtained at lower reaction times (1, 3, and 6 h) were slightly superior than the reaction system with MeCN.

In addition to the high activity demonstrated by MOF-808 in this ring-opening reaction of cyclohexene oxide with aniline, it revealed interesting selectivity, originating N-phenylcyclohexamine (88.5%) as the main product and as a secondary product, *trans*-1,2-dietoxycyclohexene (4.6%) (Figure 9b). In fact, the results obtained for the ring-opening reactions of styrene oxide and cyclohexene oxide with aniline confirm the good efficiency of MOF-808 as a catalyst in this type of reaction, both in terms of activity and selectivity.

### 3.3. Catalyst Reutilization and Stability

**Figure 10:**
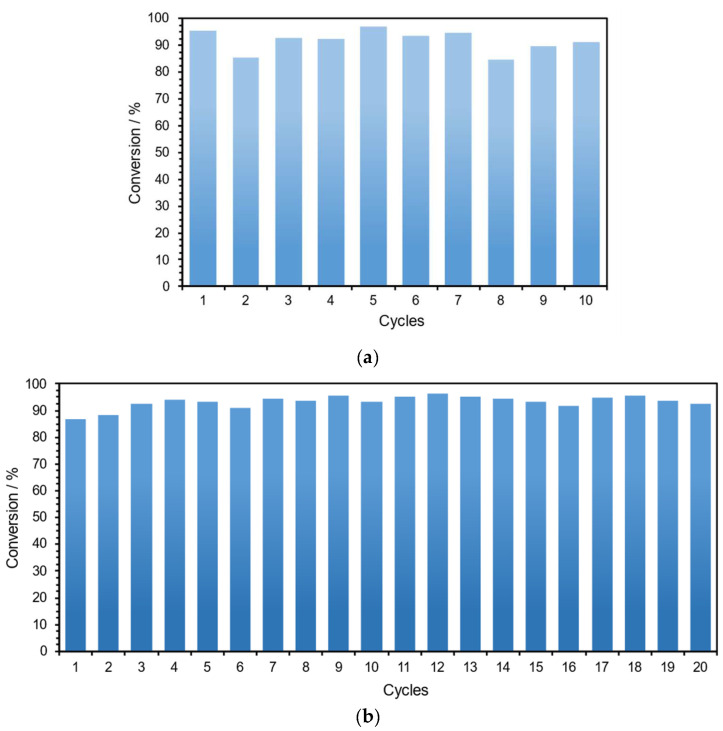
Results obtained in the recycling study (**a**) and for the reusing tests (**b**) of the catalyst MOF-808, obtained after 24 h of the ring-opening reaction of styrene oxide (1 mmol) with aniline (0.9 mmol), using 0.5 mL of EtOH as solvent, at 70 °C.

**Figure 11:**
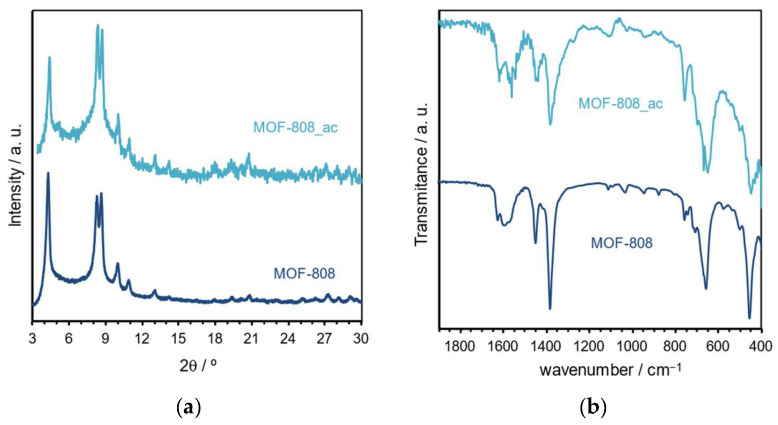
(**a**) Powder XRD patterns and (**b**) FT-IR spectra of MOF-808 before and after catalytic application (sample recovered after 20 consecutive cycles of reutilization).

The capacity of recycling and reutilizing MOF-808 in several catalytic cycles of ring-opening reactions of epoxides was evaluated using styrene oxide (1 mmol), aniline (0.9 mmol), and 0.5 mL EtOH at 70 °C. Figure 10 presents the results obtained after performing the various consecutive reactions for 24 h. In the recycling procedure, after each reaction cycle, MOF-808 was recovered by centrifugation, washed carefully, and dried. To perform a consecutive reaction cycle, the recovered and treated catalyst was weighed, and all the experimental conditions were adjusted and maintained, i.e., the quantities of the reagents and volume of EtOH. For the recycling study, it was possible to use the MOF-808 catalyst for ten consecutive cycles without any relevant loss of catalytic efficiency (the conversion was maintained around 90% with a slight decrease after the seventh cycle) (Figure 10a). It was not possible to evaluate more recycle cycles because of experimental limitations, namely, the amount of catalyst that was slightly reduced after each cycle because of some losses in the separation and cleaning processes. To overcome these experimental limitations, the reutilization ability of MOF-808 was further evaluated. In this procedure, at each new reuse cycle, the solid catalyst was maintained in the reaction vessel (without any additional treatment), and the same amount of styrene oxide and aniline and the same volume of solvent was added to proceed with the reaction under the same experimental conditions for all the reuse cycles. Notably, MOF-808 also revealed high catalytic efficiency during 20 consecutive reutilization cycles for the reaction of the ring opening of styrene oxide with aniline (Figure 10b). The conversion was maintained at around 90% without a significant loss of activity during the 20 reaction cycles. Based on the recycling and reuse behavior of the MOF-808 catalyst during the high number of cycles, it is possible to predict the high structural stability of this material under the aminolysis reaction. To investigate the structural stability of the catalyst after catalytic use, this was characterized by several techniques. The FT-IR spectroscopy and powder XRD analysis were performed of the catalyst after 20 reusing cycles (Figure 11). The powder XRD pattern of the recovered catalyst revealed the same crystalline structure of the initial MOF-808, eventually showing a slight reduction in its crystallinity (Figure 11a). This evidence is supported by the FT~IR spectrum being identical to that of the pristine MOF-808 (Figure 11b). This complementary characterization confirms that the structure of the MOF material remains unchanged after reuse. In fact, these results demonstrated that MOF-808 is an effective heterogeneous catalyst, with high efficiency and notable reusability in the ring-opening reaction of epoxides with amines.

### 3.4. Comparison with Reported Works

Only a limited number of examples can be found in the literature reporting the application of MOF structures as catalysts for the aminolysis of epoxides with aniline (Table 2). The first work was presented by Jiang et al. in 2008, who used Cu-MOF as a heterogeneous catalyst at room temperature under a solvent-free system [47]. Under these conditions, only 32% of cyclohexene oxide was converted after 4 h, using a high excess of aniline (21 mmol instead of 0.9 mmol as used in this work). The authors state that the low conversion is mainly due to diffusion limitations in the micropores of this Cu-MOF. In 2010, Garcia et al. used Fe-BTC MOF as a catalyst for the ring opening of styrene oxide using acetonitrile as solvent (10 mL). After 24 h, a moderate yield was obtained (72%) with a substrate/catalyst ratio of 410 [48]. Eight years later, Anbu et al. used the same Fe-BTC MOF and copper Cu_3_(BTC)_2_ catalysts for styrene oxide aminolysis, under a solvent-free system, but they still used a higher catalyst excess than Gracia et al. used in 2010 [49]. The conversion obtained under these conditions was not improved compared to the previous work of Garcia et al. (Table 1). Previously, in 2010, Kumar studied the same reactions using [Co^3+^-Ln^3+^] heterobimetallic one-dimensional zigzag coordination polymers as heterogeneous catalysts [50]. High yields to produce β-amino alcohols were obtained using a near equimolar ratio of substrates and aniline, at room temperature and under solvent-free conditions. Further, in this study, the solid catalyst was reused for only three catalytic cycles and some loss of activity could be observed mainly for the aminolysis reaction with styrene oxide. This is a considerable advantage of this system, mainly when expensive lanthanide metals are used in a high amount of catalyst. In 2017, our research group investigated the catalytic activity of MIL-101(Fe) for the aminolysis of cyclohexene oxide [28]. At this time, MeCN was used as a solvent, and a high amount of catalyst was needed to achieve high conversion. In this work, it was also verified that the presence of amine functional groups in the MOF structure did not affect its catalytic performance. To analyze the importance of Fe in the styrene oxide aminolysis reaction, MIL-101(Fe)-NH_2_ was used as a support to incorporate iron-monosubstituted polyoxometalate [PW_11_Fe(H_2_O)O_39_]^5−^ (abbreviated as PW_11_Fe) [36]. In this case, the composite catalyst presented an appreciable increase in catalytic activity with the near complete aminolysis of styrene oxide after 1 h of reaction, instead of 22% obtained by the isolated support MIL-101(Fe)-NH_2_. Comparing the results obtained previously (Table 1) with those obtained in the present work, it is possible to observe that the enormous advantage of using MOF-808 and UiO-66-NH_2_ is the almost negligible amount of solid MOF catalyst necessary to achieve a near complete aminolysis reaction using a system with a low amount of a sustainable solvent (0.5 mL EtOH). Another advance present in this work was the high number of heterogenous catalyst reusing cycles (no extra solvents or thermic treatments were needed) that were possible to perform without loss of catalyst activity.

## 4. Concluding Remarks

The incessant demand to develop chemical processes as sustainable as possible, aiming their application at a large scale without harming the environment, is a current research issue of extreme importance. In particular, the use of greener solvents to replace toxic solvents, which are pollutants and harmful, in the preparation of chemical materials such as MOFs, and the development of these materials as heterogeneous catalysts that are active and reusable, are important factors that have been considered along with the research work reported in this manuscript.

The Zr-based material MOF-808, a crystalline porous coordination polymer, was prepared by a straightforward room temperature procedure using water as a solvent, in contrast to the following synthesis method initially developed and widely used to prepare this MOF: the solvothermal method with DMF at a temperature of 120 °C and a time reaction of at least 24 h. This MOF-808 prepared by a much more sustainable method revealed identical structural features reported in the literature for the parent material isolated by the typical solvothermal method, as confirmed by powder XRD, vibrational (FT-IR and FT-Raman) spectroscopies, TGA, and SEM/EDS. Furthermore, this material was evaluated as a heterogeneous catalyst for ring-opening reactions of epoxides with amines for the first time. In particular, the reactions of styrene oxide or cyclohexene oxide with aniline in the presence of MOF-808 have been studied using MeCN or EtOH solvents. This MOF material revealed high activity and selectivity for both the ring-opening reactions of styrene oxide and cyclohexene oxide using a very small amount of solvent (0.5 mL of EtOH) and catalyst (an equivalent amount containing 1 μmol of Zr). Furthermore, it demonstrated notable recycling and reuse ability since MOF-808 was used for 10 consecutive recycling cycles and 20 successive reusing cycles in the reaction of styrene oxide (in EtOH) without significative loss of efficiency and maintaining its structural stability.

The investigation reported in this manuscript is a clear demonstration of the possibility of enhancing the sustainability of the chemical processes either in the preparation of functional MOFs or in their application as effective heterogeneous catalysts. Now, other MOF materials are being prepared by alternative green methods, showing their potential application as catalysts, as well as in gas separation/capture and harmful gas sensing.

## Figures and Tables

**Table 1:** Conversion of the aminolysis reaction of epoxides (styrene oxide and cyclohexene oxide) with aniline in the absence of a catalyst after 24 h of reaction (blank experiments).

	Solvent	Conversion (%)
Styrene oxide	1.5 mL EtOH	10%
	0.5 mL EtOH	16%
	1.5 mL MeCN	14%
Cyclohexane oxide	1.5 mL EtOH	10%
	0.5 mL EtOH	11%
	1.5 mL MeCN	13%

**Table 2:** Reported works for the aminolysis reaction of epoxides (styrene oxide and cyclohexene oxide) with aniline catalyzed by MOF structures.

Catalyst	Epoxide	Epoxide/Aniline (mmol)	Catalyst Amount	Solvent	T (°C)	Time (h)	Conv. (%)	Refs.
Cu-MOF	CyclohexOx	1/21	0.11 mmol	no	rt	4	32	[47]
Fe-BTC	StyrOx	41.6/41.6	150 mg	MeCN	80	24	72	[48]
[Co^3+^-Ln^3+^] ^a^	CyclohexOx	0.98/1.18	49 μmol	No	rt	4	98	[50]
[Co^3+^-Ln^3+^] ^a^	StyrOx	0.87/1	44 μmol	No	rt	4	84	[50]
MIL-101(Fe)	CyclohexOx	1/0.9	55 μmol	MeCN	80	24	87	[28]
MIL-101(Fe)-NH_2_	CyclohexOx	1/0.9	55 μmol	MeCN	80	24	86	[28]
PW_11_Fe@MIL-101(Fe)	StyrOx	1/0.9	50 μmol	MeCN	80	1	97	[36]
MIL-101(Cr)	StyrOx	1/0.9	50 μmol	MeCN	80	5	0	[36]
Cu_3_(BTC)_2_	StyrOx	0.3/0.25	25 mg	No	60	24	75	[49]
Fe-BTC	StyrOx	0.3/0.25	25 mg	no	60	24	77	[49]
MOF-808	StyrOx	1/0.9	1 μmol	EtOH	70	6	83	here
UiO-66-NH_2_	StyrOx	1/0.9	1 μmol	EtOH	70	24	88	here
MOF-808	CyclohexOx	1/0.9	1 μmol	EtOH	70	24	88	here

^a^ the catalyst is [Co^3+^-Ln^3+^] coordination polymer with Ln = Eu or Tb; rt = room temperature.

## Data Availability

The data are not publicly available due to privacy.
